# Evaluating whether direct-to-consumer marketing can increase demand for evidence-based practice among parents of adolescents with substance use disorders: rationale and protocol

**DOI:** 10.1186/s13722-015-0028-3

**Published:** 2015-02-10

**Authors:** Sara J Becker

**Affiliations:** Department of Behavioral and Social Sciences, Center for Alcohol and Addictions Studies, Brown University, 121 South Main Street, Providence, RI 02903 USA; Department of Psychiatry and Human Behavior, Warren Alpert School of Medicine, Brown University, 222 Richmond Street, Providence, RI 02903 USA

**Keywords:** Direct-to-consumer, Marketing, Dissemination, Adolescents, Substance use disorders

## Abstract

**Background:**

Fewer than one in 10 adolescents with substance use disorders (ASUDs) will receive specialty treatment, and even fewer will receive treatment designated as evidence-based practice (EBP). Traditional efforts to increase the utilization of EBP by ASUDs typically focus on practitioners—either in substance use clinics or allied health settings. Direct-to-consumer (DTC) marketing that directly targets parents of ASUDs represents a potentially complementary paradigm that has yet to be evaluated. The current study is the first to evaluate the relevance of a well-established marketing framework (the Marketing Mix) and measurement approach (measurement of perceived service quality [PSQ]) with parents of ASUDs in need of treatment.

**Methods/design:**

A mixed-methods design is employed across three study phases, consistent with well-established methods used in the field of marketing science. Phase 1 consists of formative qualitative research with parents (and a supplementary sample of adolescents) in order to evaluate and potentially adapt a conceptual framework (Marketing Mix) and measure of PSQ. Phase 2 is a targeted survey of ASUD parents to elucidate their marketing preferences, using the adapted Marketing Mix framework, and to establish the psychometric properties of the PSQ measure. The survey will also gather data on parents’ preferences for different targeted marketing messages. Phase 3 is a two-group randomized controlled trial comparing the effectiveness of targeted marketing messages versus standard clinical information. Key outcomes will include parents’ ratings of PSQ (using the new measure), behavioral intentions to seek out information about EBP, and actual information-seeking behavior.

**Discussion:**

The current study will inform the field whether a well-established marketing framework and measurement approach can be used to increase demand for EBP among parents of ASUDs. Results of this study will have the potential to immediately inform DTC marketing efforts by professional organizations, federal agencies, clinicians, and clinical researchers.

## Background

The dissemination and implementation (D&I) of effective behavioral interventions for adolescents with substance use disorders (ASUDs) has been recognized as a critical initiative by legislators, insurers, funding agencies, researchers, and clinicians alike [1]. Relative to adults with substance use disorders, ASUDs have more rapid progression from first use to a diagnosable disorder, more binge episodes, and more co-occurring problems [2,3]. When left untreated or ineffectively treated, ASUDs often have persistent problems into adulthood, increasing the risk of a range of negative long-term outcomes, including school failure, accidents, criminal involvement, unintended pregnancy, and even death [4]. Fortunately, recent systematic reviews and meta-analyses have identified several outpatient interventions that have consistently been associated with reductions in adolescent substance use in randomized controlled trials (RCTs) [5-8]. These interventions are commonly recognized as evidence-based practices (EBPs) and include cognitive behavioral therapy models (both individual and group), family therapy models (both behavioral and ecological), and motivational interviewing. Considering that over 80 percent of ASUDs seek treatment in outpatient settings [9], increasing the utilization of these EBP models represents a significant priority for our field and for our nation’s public health.

### Prior D&I efforts

Prior efforts to increase EBP utilization can be viewed as targeting two related, yet distinct, gaps. The first is the “research-to-practice” or “evidence-to-practice” gap and pertains to the disparity between those treatment models with the greatest evidentiary support and those delivered in practice [10,11]. Attempts to close this gap have predominantly targeted individual clinicians and organizational systems as deliverers of EBP, with the goal of increasing the supply of EBP in community settings. Over the past two decades, recognition of the myriad challenges bridging the research-to-practice gap has led to an exponential increase in publications on how to effectively transfer EBP to community treatment providers, with topics including: (a) development of conceptual models and frameworks to guide EBP transfer [12]; (b) identification of barriers and facilitators to adapting EBP [13,14]; (c) assessment of provider and agency interest in EBP [15]; (d) evaluation of training and roll-out strategies [16-18]; and (e) effectiveness studies testing EBP delivered under “real-world” circumstances [19]. Additionally, federal agencies have invested significant resources toward promoting the delivery of EBP in routine clinical practice, such as the Substance Abuse and Mental Health Services Administration-funded network of Addiction Technology Transfer Centers and the National Institute on Drug Abuse-funded Clinical Trials Network.

The second gap is the “treatment-seeking” or “unmet need” gap and refers to the chasm between those individuals who need treatment and those who actually seek it [20]. Despite the fact that only about 9 percent of ASUDs receive any form of specialty treatment [21], efforts to close this gap have been the focus of significantly less research and funding. The principal approach for targeting the treatment-seeking gap has been a focus on training allied health professionals and “gatekeepers” to improve the identification of ASUDs. One common strategy has been to integrate ASUD assessment and brief intervention into a variety of allied health-care settings, including primary care offices, emergency departments, schools, court systems, and detention centers/prisons. Integration efforts have been promoted through programs such as universal ASUD screening [22], enhanced training in ASUD assessment [23], and co-located ASUD treatments [24]. Specific approaches to promote the integration of ASUD services into routine practice include, but are not limited to, Screening, Brief Intervention, and Referral to Treatment [22] and assertive outreach models [25].

An important limitation of prior D&I research in the ASUD field is that it has primarily focused on practitioners—both in specialty and allied health services—as the target audience of outreach efforts. With the exception of universal screening initiatives, these efforts have focused on increasing the supply of EBP in the community, without considering whether parents of ASUDs will demand the services provided. Consequently, prior D&I initiatives are most likely to benefit ASUDs who seek services of their own volition or who come into contact with the treatment system due to a concurrent mental health, behavioral, or legal problem. As explicated by Ozechowski and Waldron [25], this approach does not address approximately 50 percent of ASUDs who do not have concurrent problems, nor does it address well-documented barriers to seeking treatment, such as lack of knowledge about treatment, lack of motivation for treatment, and a belief that problems can be solved without help. Thus, there is significant need to develop new strategies to increase the demand for EBP by parents of ASUDs.

### Direct-to-Customer (DTC) marketing: a complementary approach

**Figure 1:**
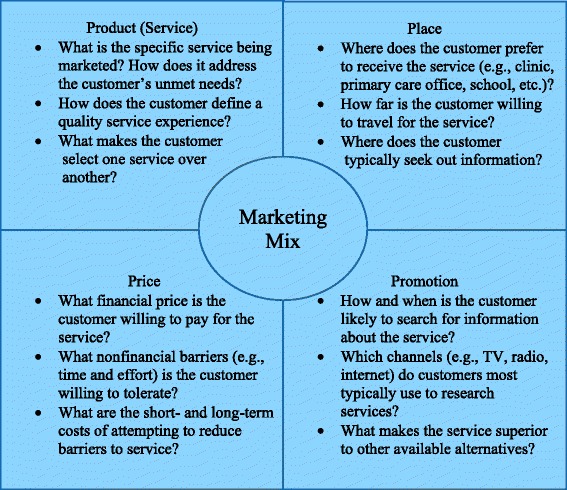
**Marketing mix elements and key questions.**

DTC marketing, defined as marketing targeted directly to treatment customers, represents a complementary paradigm to traditional D&I models. In contrast to traditional approaches that attempt to “push” EBP to patients through treatment providers, a DTC approach attempts to increase awareness of EBP so that customers will request it and “pull” it through the system. A well-established framework in the marketing literature is the Marketing Mix [26], otherwise known as the “4 P’s,” which refer to: Product (or Service), Price, Place, and Promotion. As presented in Figure 1, each component of the Marketing Mix reflects a key set of considerations that need to be addressed before marketing a service. Recent manuscripts have considered how the Marketing Mix might apply to the delivery of mental health and substance use treatment [27,28], but the relevance of the Marketing Mix has never been assessed with actual treatment customers. Reflecting this gap in our knowledge base, this study aims to test whether the Marketing Mix can be used as a guiding framework to develop effective DTC marketing messages for parents of ASUDs.

Although all four elements of the Marketing Mix are important, scholars in the field of services marketing (the marketing of professional services) have argued that the Product (or Service) component is especially vital, because of the need to understand how customers define a quality service experience. In a widely cited article, Berry, Parasuraman, and Zeithaml (pg. 35) asserted that “service quality has become the great differentiator, the most powerful competitive weapon most service organizations possess,” and they further argue that “it is the customer’s definition of quality, not management that counts” [29]. Services are significantly more difficult to evaluate objectively than products, because they are intangible, heterogeneous over time and across providers, and difficult to reproduce; consequently, customers’ *subjective* perceptions of quality become critically important predictors of whether customers will actually seek out the service [29,30]. Over the past 25 years, the Marketing Science Institute has supported extensive research on customer perceptions of service quality [29-37]. Formative qualitative research in several industry sectors [31,34,38] led to the development of SERVQUAL [39,40] a questionnaire that evaluates perceived service quality (PSQ) across five dimensions: Reliability, Responsiveness, Assurance, Empathy, and Tangibles. The SERVQUAL is widely accepted as the gold-standard measure of PSQ, having been used in over 70 service industries across 17 countries [26,41]. Multiple other measures of PSQ have also been developed and used in marketing research [42,43].

### Benefits of measuring perceived service quality

Prior marketing studies have demonstrated three major advantages of using measures like the SERVQUAL to evaluate PSQ. First, use of PSQ measures can establish the service quality attributes that most strongly predict behavioral intentions with regard to service selection and loyalty [33,37]. Such knowledge can potentially be used to develop targeted marketing messages to increase customer demand for new services and to devise concrete strategies to increase customer satisfaction [30]. Second, repeated administration of PSQ instruments can be used to evaluate whether specific marketing initiatives are effective in changing customers’ opinions of service [26,30]. Finally, service providers can periodically use a PSQ questionnaire to ensure that customers receive consistent, high-quality service, which can then be used to support specific marketing claims [26].

To date, PSQ measures have been used to evaluate health services in a range of settings across multiple countries, including nursing homes, hospitals, outpatient medical clinics, and dental clinics [44-50]. However, only one prior study has used a PSQ questionnaire to evaluate mental health services [50], and no studies have used one to evaluate SUD services. Moreover, an examination of prior studies reveal a number of modifications to extant PSQ questionnaires to increase their relevance to health services. Focusing on the SERVQUAL, modifications in prior health-care studies in the United States have included removing items [45,50], revising items [45,49,50], and adding new items [49,51]. These modifications have been associated with changes to the SERVQUAL’s psychometric properties and structure, with some studies finding that the SERVQUAL had three or four dimensions [52] and others finding six or more dimensions [49,51,53]. New quality dimensions found in more than one study have included “caring”, “empathy”, and “provider competence”.

An alternative to idiosyncratic, study-specific modifications is the development of a refined PSQ measure specifically for the assessment of ASUD services. There is a precedent for this approach in the marketing literature. Approximately 10 years ago, the SERVQUAL was frequently applied to assess ecommerce service transactions, despite a number of theoretical differences between standard services and online services [54]. The SERVQUAL developers therefore conducted formative qualitative research and customer surveys, resulting in a new measure (the E-S-QUAL) with different items and a new dimensional structure [55]. Following this precedent, the current study proposes to use qualitative research and survey methods to create a new PSQ questionnaire for use with ASUD behavioral treatments.

### Specific aims and study phases

As depicted in Table 1, this study has three phases that address four specific aims. Phase 1 consists of formative qualitative research in order to evaluate and potentially adapt a conceptual model (the Marketing Mix) and quantitative measure (a PSQ measure based on the SERVQUAL) to evaluate treatment quality (**Aim 1**). Phase 2 is a web-based survey of ASUD parents to establish the psychometric properties of the PSQ measure (**Aim 2**) and to elucidate the marketing preferences of the target population (using the adapted Marketing Mix framework, **Aim 3**). Phase 3 is a two-group, web-based RCT comparing the effectiveness of targeted marketing messages versus standard clinical information in prompting parents of ASUDs to seek information about EBP (**Aim 4**). The RCT in Phase 3 is designed to test the hypotheses that relative to parents who view standard clinical information, parents of ASUDs who view targeted marketing messages will demonstrate: 1) greater PSQ ratings (as assessed using the new measure); 2) greater reported behavioral intentions to seek out EBP; and 3) greater likelihood of actually seeking out information about EBP.

**Table 1:** **Timeline of research activities by study phase**

**Phase**		**Year 1**				**Year 2**				**Year 3**				**Year 4**				**Year 5**			
1	Focus groups and structured interviews	X	X	X	X																
	Thematic coding and analysis				X	X	X														
	Adaptation of Marketing Mix and PSQ **(AIM 1)**							X	X												
2	Survey design, sampling, and implementation									X	X	X	X								
	Survey analysis and PSQ validation **(AIMS 2–3)**												X	X	X						
3	Randomized trial of targeted marketing **(AIM 4)**															X	X	X			
	Quantitative analysis and results dissemination																		X	X	X

## Methods/design

### Design considerations

Four key decisions were made related to study design. First, this study focuses on EBP as a modality, rather than on a specific therapy model. In the pharmaceutical industry, investment in DTC marketing increased by 330 percent from 1996 to 2005 [56]. Analysis of this investment has unequivocally established that DTC marketing can increase demand for both individual products (e.g., Prozac) and entire classes of products (e.g., selective-serotonin reuptake inhibitors) [56]. Hence, developing targeted marketing has the potential to stimulate customer demand not only for individual therapy models, but also for EBP as a class of treatment. Second, this study identifies parents of ASUDs as the target population instead of ASUDs themselves. DTC marketing principles are most relevant for customers who select, initiate, and finance a service [57]. Whereas in many disciplines the customer who purchases the service is also the consumer who uses the service, this is rarely the case for ASUD treatment. It has been established that ASUDs rarely seek treatment of their own volition [58] and that parents are more likely than youth to make decisions about treatment selection [59,60]. This study therefore prioritizes parent (customer) feedback and augments it with ASUDs (consumer) feedback in the formative qualitative research phase. Third, study eligibility is based on parental concern about the teen’s substance use. Specifically, parent report of the teen’s behavior must meet the clinical cutoff on a substance use screen. Although parents often underestimate ASUD severity [61], this study aims to understand factors that influence utilization of treatment. Because parents are more likely than youth to select treatment, parent impressions are viewed as more likely to drive demand for service than objectively measured or self-reported levels of adolescent use. Finally, recruitment targets parents of ASUDs who have either not yet engaged in treatment (e.g., high risk, nontreatment seeking) or who have presented to treatment voluntarily (e.g., not mandated by juvenile justice), as these parents are likely to have the most choice in service selection.

### Participants

Parents of ASUDs, the target population for this study, must meet the following criteria: 1) primary legal guardian of a teenager aged 12 to 17 years; 2) able to speak and read in English; 3) report significant concern about their teen’s substance use, as determined by their responses to a brief screening tool (see Measures). Adolescents automatically qualify if their parents meet the aforementioned criteria. Exclusion criteria are minimal to enhance generalizability and include factors that would preclude participation in a 1–2 hour study session (e.g., acute psychosis, insufficient cognitive capacity, etc.). In accordance with institutional IRB approval, parents provide informed consent, and adolescents (in Phase 1 only) provide written assent.

In Phase 1, participants were recruited from treatment sites in the northeast region of the United States that provide services for ASUDs across the full continuum of care: one primary care clinic, one adolescent mental health clinic, one ASUD outpatient program, and one ASUD residential treatment program. In Phases 2 and 3, recruitment at these clinics will be supplemented with online recruitment methods. Participant demographics are expected to be representative of the population in the northeast region. Based on regional demographics and enrollment rates in prior studies, the sample is expected to be 70 percent white, 8 percent African American, 1 percent Asian American, 1 percent American Indian, and 19 percent “other” or multiracial. Ethnic composition is expected to be 33 percent Hispanic. Parents are expected to be at least 80 percent female, whereas adolescents are expected to be 60 percent male.

### Measures

In all three study phases, participants complete three brief measures: 1) a basic socio-demographic form; 2) a brief screen about parental substance use (NIDA-Quick Screener [62]; and 3) the Global Appraisal of Individual Needs–Short Screener (GAIN-SS, [63]). The GAIN-SS is a well-validated screening tool that consists of four 5-item screeners: substance use disorders, internalizing problems, externalizing problems, and criminal behavior. Each screener has demonstrated internal consistency, excellent sensitivity (over 90%) for identifying people with a disorder, and excellent specificity (over 95%) for ruling out people without a disorder [63]. A positive response to one or more items in each sub-screener indicates a moderate likelihood of a diagnosis or significant problems. Prior to enrollment, parent responses to the substance use disorder screener are used to determine eligibility. After enrollment, responses to all four screeners are used to track the level of adolescent substance use and co-occurring problems within the sample.

### Procedures: study phase 1 (Specific Aim 1) — months 1–22

Phase 1 consisted of separate focus groups and structured interviews with ASUDs (n = 23) and their parents (n = 29). Data from this phase addresses study Aim 1 to adapt the Marketing Mix framework and to develop a PSQ measure to ensure their relevance to the marketing of ASUD treatment.

### Focus groups and interviews

Participants participated in either a focus group or individual interview, depending on participant preference and recruitment flow. Focus groups enrolled four to six participants and lasted 75–90 minutes, whereas individual interviews lasted 45–60 minutes. Focus groups were homogeneous with regard to level of care and conducted separately for parents and ASUDs. A trained facilitator with over 10 years of qualitative experience led the discussions, and a co-facilitator attended the focus groups to take notes. All discussions followed a semistructured agenda covering fundamental marketing concepts [26], including the four elements of the Marketing Mix, ideal treatment experience, and dimensions of PSQ.

### Qualitative analysis

Audio files from focus groups and interviews were transcribed verbatim by the co-facilitator and cleaned by the lead facilitator. Transcripts were entered into NVivo, a qualitative data management program. Parent and adolescent data will be analyzed separately using standard qualitative analysis techniques, including open coding, axial coding, marginal remarks, and memo writing [64]. A list of thematic codes will be developed, beginning with codes based on the agendas, and augmented with codes emerging from the data. Codes for parent data will be identified first and supplemented by unique codes in ASUD data. Two independent coders will double-code 25 percent of transcripts, aiming for an overall kappa over .60.

A modified grounded theory approach will be used to organize themes arising from discussions to assess the relevance of the Marketing Mix and to determine dimensions of PSQ [64]. Based on prior research on service quality [30,41,49,53,65] and barriers to adolescent utilization of EBP [59,60,66,67], we expect that dimensions of PSQ for ASUD treatment may include reliability, responsiveness, access, competence, tangibles, courtesy, integrity, attentiveness, communication, credibility, comfort, empathy, caring, availability, security, assurance/trust, affordability, flexibility, personalization, treatment relevance, treatment compatibility, treatment alliance, and convenience.

Within each of the identified dimensions, verbatim comments will be used to create an initial pool of items for a PSQ measure. Participant comments will also be used to create a draft pool of targeted marketing messages highlighting the benefits of EBP. Messages will be developed using participants’ comments about the elements of an ideal service experience, including references to service attributes that most strongly influence treatment selection. As an example, if parents report that therapist flexibility is the most important service attribute, the statement would emphasize that EBP can be tailored to individual teens using specific words from parents’ verbatim comments. Imbedding valued service attributes into marketing materials has been shown to increase customers’ intentions to seek out a specific service [68].

### Cognitive interviews

Cognitive interviews, a qualitative approach to questionnaire modification, will be conducted with an additional six ASUDs and six ASUD parents to assess the relevance and clarity of the adapted Marketing Mix and PSQ. Interviews will use the respondent debriefing technique [69] and last 60–75 minutes. Each interview will consist of administration of the PSQ measure, followed by a series of questions about the clarity and rationale of directions, the perceived meaning of each item, the appropriateness of response options, and the overall relevance of the measure. Targeted marketing messages about EBP will be tested using the same methods. Interviews will be recorded with DVR and transcribed verbatim. Feedback will be aggregated by questionnaire component (e.g., directions, items, response options) to inform revisions.

### Procedures: study phase 2 (Specific Aims 2 and 3) — months 23–42

Phase 2 uses a survey of parents of ASUDs (n = 400) to serve two goals: 1) establish the psychometric properties of the PSQ, and 2) elucidate the marketing preferences of ASUD parents using the Marketing Mix framework. Surveys are often used in marketing research to test initial qualitative findings using direct questions in larger, more representative samples [26].

### Survey construction

In addition to containing the measures used in Phase 1, the survey will contain the PSQ and a series of questions about service preferences (about 40 items). Items about service preferences will be developed based on feedback from Phase 1 and will cover each element of the Marketing Mix framework. An example of a question from the “Place” component might be a multiple-choice item indicating where parents prefer to receive their information about ASUD treatment, with response options based on the data from Phase 1. Also, parents will be asked to rank-order their preferred targeted marketing messages from Phase 1.

### Sampling considerations

Several sampling frames were considered for the survey, each of which had benefits and tradeoffs. First, a nonprobabilistic (or nonrandom) online survey was considered, as this approach is commonly used in marketing research [70]. As noted in Zeithaml et al.’s best-selling services marketing textbook [26], the benefits of online surveys include: broader geographic representation; enhanced accuracy and completeness of answers; speed; reduced costs; and streamlined data analysis. However, this approach was ruled out due to concerns about selection bias and limited coverage of individuals without internet access [71]. Second, community-based recruitment was contemplated, as this approach is common in treatment research. Although this approach yields a nonprobabilistic sample, it protects against some of the concerns about the reach of an online sample by facilitating proactive recruitment of low-income and under-represented populations. Finally, various methods to recruit a probabilistic sample were considered, each of which would require members of the target population to have an equal and random chance of selection [72]. This approach would involve random selection of participants from a known sample of parents of ASUDs through national registries, school rosters, or other means. Although this approach would yield the largest benefits in terms of representativeness, its execution would require the largest investment of time and financial resources. As such, probabilistic sampling was deemed beyond the scope of this protocol, which was funded by a Research Career Development Award (K23) and designed to collect preliminary data sufficient to establish proof of concept.

Based on the aforementioned considerations, the survey will use multimethod, single-mode sampling, combining community-based and online recruitment. Within each method, quota sampling will be used to promote representativeness with regard to geography, race/ethnicity, gender, and parent characteristics in order to more closely approximate a probabilistic sample [72]. All participants will be administered the survey using a web-based browser to prevent confounds associated with modality. The community sample will complete the survey on a laptop during an in-person study session, while the online sample will complete the survey from a computer of their choosing. The online sample will be restricted to the six-state New England area in order to promote consistency with the community sample. Reflecting the differential level of effort required, participants in the community will receive $50, while those in the online sample will receive $25.

### Online recruitment

One-quarter to one-half of the survey participants will be recruited through the clinics used in Phase 1. Remaining participants will be recruited online, using an existing website of the American Psychological Association’s (APA) Division 53 (Society of Clinical Child and Adolescent Psychology). The website (http://www.effectivechildtherapy.com) contains pages specifically designed to educate parents about EBP for adolescents with mental health and/or substance use problems. Banners will be placed on the Substance Abuse and Dependence page, along with pages for two conditions that are highly comorbid among ASUDs: Disruptive Behavior Problems and Depression and Related Disorders. Based upon webpage usage statistics at the time of this protocol submission, these three pages respectively received an average of 108, 712, and 354 unique hits per month. Combining these data with national estimates of internet usage [73] and prevalence rates of co-occurring problems among ASUDs [74-76] leads to the conservative estimate that approximately 29–40 webpage visitors per month likely will meet study eligibility criteria. Using another conservative estimate that only 50 percent of visitors will likely participate (due to repeat visitors, ineligibility, and lack of interest), this study will set a recruitment target of four parents per week. Quota sampling will be used to promote equivalence across the online and community-based samples on key demographic (e.g., age, race/ethnicity, socio-economic status, region) and clinical variables (parental substance use, number of concerns about teen substance use endorsed on the screener); if sample differences are detected across the two sampling frames, a propensity weighting approach will be used to balance the samples [77] prior to merging the data.

### Survey analysis

Descriptive statistics will be generated to summarize responses for the market research questions. Additional analyses will be conducted to test the psychometric properties of the new PSQ, including its factor structure, internal consistency, and validity (criterion, convergent, and discriminant). Items with a high proportion of missing data will be eliminated to create a more parsimonious scale. The following analyses are planned: *Factor Structure*. The target sample of 400 and expected questionnaire length of 20 items will enable analyses to be conducted using both Exploratory Factor Analysis (EFA) and Confirmatory Factor Analysis (CFA). EFA will be conducted using principal component analysis as the extraction method and promax as the rotation method. CFA will be conducted to further assess the scale’s structure. Model fit will be evaluated using conventional-fit indices, including RMSEA, CFI, and chi-square [78]. *Reliability*. Internal consistency for each scale will be calculated using Cronbach’s alpha, with values exceeding .7 viewed as evidence of high internal consistency [79]. *Criterion Validity*. Consistent with the initial SERVQUAL validation [39], criterion validity will be tested using global assessments of service expectations on a 10-point scale (1 = poor, 10 = excellent). *Convergent Validity*. Loading of scale items on their corresponding factors will be examined to assess convergent validity. *Discriminant Validity*. Discriminant validity will be tested by constraining each correlation between pairs of factors (one at a time) to unity in the measurement model and repeating the CFA. If the constrained CFA increases chi-square, it will support the distinctiveness of each scale’s component dimensions.

### Procedures: study phase 3 (Specific Aim 4) — months 43–60

Phase 3 will use a two-group RCT (n = 160) to evaluate the effectiveness of targeted marketing versus standard clinical information in increasing parent impressions of PSQ and persuading parents of ASUDs to seek out information about EBP. Study hypotheses will be tested using the PSQ, which was adapted and validated in Phases 1–2, as well as behavioral indicators of information-seeking. Recruitment will occur using the same eligibility criteria and multimethod, single-mode approach as Phase 2.

### Sample size and power

One meta-analysis of the effect size (ES) of marketing campaigns on the proportion of customers inquiring about or purchasing a service found an average ES of .20 across 133 studies [80]. Of note, only one of the 133 studies tested marketing for a health-care service, and this study found a higher ES of .30. This ES is consistent with results of a recent quasi-experimental study comparing the effectiveness of a DTC campaign versus standard clinical guidelines in predicting cervical cancer screenings (d = .26) [81]. Assuming that awareness of the services marketed in prior studies was higher than current awareness of EBP, targeted marketing has the potential to produce larger ES in the current study. The target sample of 160 (n = 80 per group) will provide 71 percent power to detect an ES of .4; 88 percent for an ES of .5; and 96 percent for an ES of .6 [82]. Thus, the proposed sample size will be adequate to detect a medium ES, which is appropriate for a study that aims to provide proof of concept.

### Randomization

A computer-generated urn randomization procedure will be used to retain random allocation, while balancing relative probabilities of assignment to treatment groups (urns). Randomization will occur after participants complete the eligibility screening and prior to the manipulation. Variables added to the urn will include both parent (e.g., gender, race/ethnicity, parent substance use) and ASUD characteristics (e.g., number of concerns endorsed on the substance use screener, history of substance use treatment).

Parents will be randomized to one of two conditions: targeted marketing or standard clinical information. Parents in the marketing condition will be presented with the descriptions of EBP that were rated as most appealing in Phase 2, whereas parents in the standard information condition will review descriptions of EBP created for parents by leading scientific organizations in our field such as the National Institute on Drug Abuse (NIDA), Division 53 of The Society of Clinical Child and Adolescent Psychology (APA), and the Substance Abuse and Mental Health Services Administration. A sample standard clinical information message (from the APA Division 53 website) is: *Some treatment approaches have a strong backing in scientific evidence and other treatments have less evidence supporting them. Therapists who use treatments based on science use what is called “evidence-based practice…”* Messages will be balanced to ensure comparable number of words.

### Data collection and measures

The trial and quantitative data collection will be administered with Ilume software, a program designed for research surveys that is frequently used by the investigative team and that minimizes the likelihood of missing data. *Baseline*. In addition to the core set of measures, parents will answer questions assessing EBP attitudes, awareness, prior use, and intent to use (based on survey items from Phase 2). Parents will then be presented with one of two descriptions of EBP, depending on their random assignment. *Post-intervention*. Parents will complete Likert-scale ratings of the presented information in terms of its relevance to their teen/family, appeal, believability, ease of understanding, and amount of information conveyed. Parents will then complete the PSQ measure and a Behavioral Intentions Scale. After parents complete these measures, they will view a message thanking them for their participation. They will then see two links: 1) a link to end the study session, and 2) a link to receive more information about EBP service options for their teenager. The proportion of parents clicking the link to receive more information will be viewed as a secondary indicator of intentions to seek out EBP.

### Outcomes

Descriptive statistics will be generated to describe the data. Mean comparisons will be used to test the hypothesis that participants in the marketing condition will have greater perceptions of service (as measured by the PSQ). The hypotheses that participants will report greater behavioral intentions to use EBP and will demonstrate greater likelihood to request information about EBP will be measured by group comparisons using continuous ratings on the Behavioral Intentions Scale and a categorical indicator of whether the participant actually requested information, respectively. If group differences emerge in these basic analyses, multiple regression models will test whether the group differences persist when controlling for pre-manipulation variables such as EBP attitudes, EBP awareness, intentions to seek therapy, and readability statistics generated on the messages. For all of the hypotheses, exploratory analyses will be conducted to evaluate potential moderators and mediators. Ethnicity/race, gender, and treatment history will be tested as potential moderators of group differences, and ratings of the information (e.g., ease of understanding, believability) will be tested as potential mediators of outcome. In addition, for the hypotheses about behavioral indicators (e.g., Behavioral Intentions Scales and likelihood of requesting information), PSQ ratings will be examined as potential mediators of outcome. These analyses will be conducted to observe trends and highlight directions for further research.

## Discussion

The current study addresses an issue of importance to our field and our nation’s public health: increasing the utilization of EBP by ASUDs. While prior studies have worked to increase the utilization of EBP by targeting treatment providers, this study evaluates the relevance of DTC marketing to deliver information directly to parents of ASUDs. Qualitative data collection from ASUDs and their parents will be used to evaluate the utility of the Marketing Mix framework in developing targeted messages and to develop a new measure of PSQ, a key construct in predicting demand for a service. A subsequent targeted survey will be used to further evaluate the Marketing Mix and to validate the PSQ measure. The final phase of the study will test the hypotheses that targeted marketing messages developed using the Marketing Mix framework, which will be associated with greater perceptions of service quality (using the PSQ measure) and greater behavioral intentions to seek out information about EBP than standard clinical information. Results of this study will have the potential to immediately inform DTC marketing efforts by professional organizations, federal agencies, clinicians, and clinical researchers.

## Abbreviations

APA: American psychological association
ASUD: Adolescent substance use disorder
ASUDs: Adolescents with substance use disorders
D&I: Dissemination and implementation
DTC: Direct-to-consumer
EBP: Evidence-based practice
ES: Effect size
PSQ: Perceived service quality

## References

[1] Institute of Medicine. Bridging the Gap Between Practice and Research: Forging Partnerships with Community-Based Drug and Alcohol Treatment. Edited by Lamb S, Greenlick MR, McCarty D. Washington, DC: National Academy Press. 1998. 25101381

[2] Chan YF, Dennis ML, Funk RR (2008). Prevalence and comorbidity of major internalizing and externalizing problems among adolescents and adults presenting to substance abuse treatment. J Subst Abuse Treat.

[3] Kandel DB, Johnson JG, Bird HR, Canino G, Goodman SH, Lahey BB (1997). Psychiatric disorders associated with substance use among children and adolescents: findings from the methods for the epidemiology of child and adolescent mental disorders (MECA) study. J Abnorm Child Psychol.

[4] The National Center on Addiction and Substance Abuse at Columbia University (2011). Adolescent Substance Use: America’s #1 Public Health Problem.

[5] Waldron HB, Turner CW (2008). Evidence-based psychosocial treatments for adolescent substance abuse. J Clin Child Adolesc Psychol.

[6] Becker SJ, Curry JF (2008). Outpatient interventions for adolescent substance abuse: a quality of evidence review. J Consult Clin Psychol.

[7] Hogue A, Henderson CE, Ozechowski TJ, Robbins MS (2014). Evidence base on outpatient behavioral treatments for adolescent substance use: updates and recommendations 2007–2013. J Clin Child Adolesc Psychol.

[8] Tanner-Smith EE, Lipsey MW (2014). Brief alcohol interventions for adolescents and young adults: a systematic review and meta-analysis. J Subst Abuse Treat.

[9] Substance Abuse and Mental Health Services Administration. Office of Applied Studies (2010). Treatment Episode Data Set (TEDS). 1998–2008. National Admissions to Substance Abuse Treatment Services. DASIS Series: S-50, HHS Publication No. (SMA) 09–4471.

[10] Bero LA, Grilli R, Grimshaw JM, Harvey E, Oxman AD, Thomson MA (1998). Closing the gap between research and practice: an overview of systematic reviews of interventions to promote the implementation of research findings. BMJ.

[11] Lang ES, Wyer PC, Haynes RB (2007). Knowledge translation: closing the evidence-to-practice gap. Ann Emerg Med.

[12] Tabak RG, Khoong EC, Chambers DA, Brownson RC (2012). Bridging research and practice: models for dissemination and implementation research. Am J Prev Med.

[13] Haynes B, Haines A (1998). Barriers and bridges to evidence based clinical practice. BMJ.

[14] Miller WR, Sorensen JL, Selzer JA, Brigham GS (2006). Disseminating evidence-based practices in substance abuse treatment: a review with suggestions. J Subst Abuse Treat.

[15] McGovern MP, Fox TS, Xie H, Drake RE (2004). A survey of clinical practices and readiness to adopt evidence-based practices: dissemination research in an addiction treatment system. J Subst Abuse Treat.

[16] Beidas RS, Kendall PC (2010). Training therapists in evidence‐based practice: a critical review of studies from a systems‐contextual perspective. Clin Psychol.

[17] Miller WR, Yahne CE, Moyers TB, Martinez J, Pirritano M (2004). A randomized trial of methods to help clinicians learn motivational interviewing. J Consult Clin Psychol.

[18] Sholomskas DE, Syracuse-Siewert G, Rounsaville BJ, Ball SA, Nuro KF, Carroll KM (2005). We don’t train in vain: a dissemination trial of three strategies of training clinicians in cognitive-behavioral therapy. J Consult Clin Psychol.

[19] Wells EA, Saxon AJ, Calsyn DA, Jackson TR, Donovan DM (2010). Study results from the clinical trials network’s first 10 years: where do they lead?. J Subst Abuse Treat.

[20] Demyttenaere K, Bruffaerts R, Posada-Villa J, Gasquet I, Kovess V, Lepine JP (2004). Prevalence, severity, and unmet need for treatment of mental disorders in the world health organization world mental health surveys. JAMA.

[21] Substance Abuse and Mental Health Services Administration (2014). Results from the 2013 National Survey on Drug Use and Health: Summary of National Findings, NSDUH Series H-48, HHS Publication No. (SMA) 14–4863.

[22] Babor TF, McRee BG, Kassebaum PA, Grimaldi PL, Ahmed K, Bray J (2007). Screening, brief intervention, and referral to treatment (SBIRT): toward a public health approach to the management of substance abuse. Subst Abus.

[23] Ewan CE, Whaite A (1982). Training health professionals in substance abuse: a review. Intl J Addict.

[24] Rothman J, Rudnick D, Slifer M, Agins B, Heiner K, Birkhead G (2007). Co-located substance use treatment and HIV prevention and primary care services, New York state, 1990–2002: a model for effective service delivery to a high-risk population. J Urban Health.

[25] Ozechowski TJ, Waldron HB (2010). Assertive outreach strategies for narrowing the adolescent substance abuse treatment gap: implications for research, practice, and policy. J Behav Health Serv Res.

[26] Zeithaml VA, Bitner MJ, Gremler DD (2009). Services Marketing: Integrating Customer Focus Across the Firm.

[27] Gallo KP, Comer JS, Barlow DH (2013). Direct-to-consumer marketing of psychological treatments for anxiety disorders. J Anxiety Disord.

[28] Becker S. Direct-to-consumer marketing: a complementary approach to traditional dissemination and implementation efforts for mental health and substance abuse interventions. Clin Psychol: Science and Practice In press. 10.1111/cpsp.12086 PMC4415980 25937710

[29] Berry LL, Parasuraman A, Zeithaml VA (1988). The service-quality puzzle. Business Horizons.

[30] Parasuraman A, Zeithaml VA, Berry LL (1985). A conceptual-model of service quality and its implications for future-research. J Marketing.

[31] Berry LL, Zeithaml VA, Parasuraman A (1985). Quality counts in services, too. Business Horizons.

[32] Berry LL, Zeithaml VA, Parasuraman A (1990). Five Imperatives for improving service quality. MIT Sloan Manage Rev.

[33] Boulding W, Kalra A, Staelin R, Zeithaml VA (1993). A dynamic process model of service quality: from expectations to behavioral intentions. J Market Res.

[34] Parasuraman A, Berry LL, Zeithaml VA (1983). Service firms need marketing skills. Business Horizons.

[35] Parasuraman A, Berry LL, Zeithaml VA (1991). Understanding customer expectations of service. MIT Sloan Manage Rev.

[36] Parasuraman A, Zeithaml V, Berry LL (1993). The nature and determinants of customer expectations of service. J Acad Market Sci.

[37] Zeithaml VA, Berry LL, Parasuraman A (1996). The behavioral consequences of service quality. J Marketing.

[38] Zeithaml VA, Parasuraman A, Berry LL (1985). Problems and strategies in services marketing. J Marketing.

[39] Parasuraman A, Zeithaml VA, Berry LL (1988). SERVQUAL—a multiple-item scale for measuring consumer perceptions of service quality. J Retailing.

[40] Parasuraman A, Berry LL, Zeithaml VA (1991). Refinement and reassessment of the SERVQUAL scale. J Retailing.

[41] Ladhari R (2009). A review of twenty years of SERVQUAL research. Int J Quality Serv Sci.

[42] Brown TJ, Churchill GA, Peter JP (1993). Improving the measurement of service quality. J Retailing.

[43] Cronin JJ, Taylor SA (1994). SERVPERF versus SERVQUAL: reconciling performance-based and perceptions-minus-expectations measurement of service quality. J Marketing.

[44] Anderson E (1995). Measuring service quality in a university health clinic. Int J Health Care Qual Assur.

[45] Babakus E, Mangold WG (1992). Adapting the SERVQUAL scale to hospital services: an empirical investigation. Health Serv Res.

[46] Dean AM (1999). The applicability of SERVQUAL in different health care environments. Health Mark Q.

[47] Kaldenberg D, Becker BW, Browne BA, Browne WG (1997). Identifying service quality strengths and weaknesses using SERVQUAL: a study of dental services. Health Mark Q.

[48] Wisniewski M, Wisniewski H (2005). Measuring service quality in a hospital colposcopy clinic. Int J Health Care Qual Assur Inc Leadersh Health Serv.

[49] Reidenback RE, Sandifer-Smallwood B (1990). Exploring perceptions of hospital operations by a modified SERVQUAL approach. J Health Care Mark.

[50] Tempier R, Hepp SL, Duncan CR, Rohr B, Hachey K, Mosier K (2010). Patient-centered care in affective, non-affective, and schizoaffective groups: patients’ opinions and attitudes. Community Ment Health J.

[51] Bowers MR, Swan JE, Koehler WF (1994). What attributes determine quality and satisfaction with health care delivery?. Health Care Manage Rev.

[52] Yeşilada F, Direktör E (2010). Health care service quality: a comparison of public and private hospitals. African J Bus Manage.

[53] Ramsaran-Fowdar RR (2005). Identifying health care quality attributes. J Health Hum Serv Adm.

[54] Zeithaml VA, Parasuraman A, Malhotra A (2002). Service quality delivery through web sites: a critical review of extant knowledge. J Acad Mark Sci.

[55] Zeithaml VA, Parasuraman A, Malhotra A (2005). E-S-QUAL — A multiple-item scale for assessing electronic service quality. J Serv Res.

[56] Donohue JM, Cevasco M, Rosenthal MB (2007). A decade of direct-to-consumer advertising of prescription drugs. New Engl J Med.

[57] Gupta S, Zeithaml V (2006). Customer metrics and their impact on financial performance. Marketing Sci.

[58] Stiffman AR, Hadley-Ives E, Dore P, Polgar M, Horvath VE, Striley C (2000). Youths‘ access to mental health services: the role of providers’ training, resource connectivity, and assessment of need. Ment Health Serv Res.

[59] Kazdin AE, Holland L, Crowley M, Breton S (1997). Barriers to treatment participation scale: evaluation and validation in the context of child outpatient treatment. J Child Psychol Psychiatry.

[60] Nock MK, Ferriter C (2005). Parent management of attendance and adherence in child and adolescent therapy: a conceptual and empirical review. Clin Child Fam Psychol Rev.

[61] Center for Substance Abuse Treatment (1996). CESAR FAX, Vol 5, Issue 16, April, 29, 1996.

[62] National Institute on Drug Abuse. The NIDA Quick Screen. In Resource Guide: Screening for Drug Use in General Medical Settings. Rockville, MD: National Institute on Drug Abuse; 2012. Retrieved from http://www.drugabuse.gov/publications/resource-guide-screening-drug-use-in-general-medical-settings/nida-quick-screen.

[63] Dennis ML, Feeney T, Stevens LH (2006). Global Appraisal of Individual Needs–Short Screener (GAIN-SS): Administration and Scoring Manual for the GAIN-SS Version 2.0.1.

[64] Strauss A, Corbin J (1990). Basics of Qualitative Research: Grounded Theory Procedures and Techniques.

[65] Parasuraman A, Berry LL, Zeithaml VA (1993). More on improving service quality measurement. J Retailing.

[66] Mensinger JL, Diamond GS, Kaminer Y, Wintersteen MB (2006). Adolescent and therapist perception of barriers to outpatient substance abuse treatment. Am J Addict.

[67] Zwaanswijk M, Van der Ende J, Verhaak PF, Bensing JM, Verhulst FC (2003). Factors associated with adolescent mental health service need and utilization. J Am Acad Child Adolesc Psychiatry.

[68] Clow KE, Tripp C, Kenny JT (1996). The importance of service quality determinants in advertising a professional service: an exploratory study. J Serv Marketing.

[69] Willis GB (2005). Cognitive Interviewing: A Tool for Improving Questionnaire Design.

[70] Ilieva J, Baron S, Healey NM (2002). On-line surveys in international marketing research: pros and cons. Int J Mark Res.

[71] Schonlau M, van Soest A, Kapteyn A, Couper M (2009). Selection bias in web surveys and the use of propensity scores. Sociolog Methods Res.

[72] Doherty M. Probability versus non-probability sampling in sample surveys. The New Zealand Statistics Review 1994: 21–28.

[73] Pew Research Center (2014). Internet user demographics. In PewResearch Internet Project. Retrieved from http://www.pewinternet.org/data-trend/internet-use/latest-stats/

[74] Zeitlin H (1999). Psychiatric comorbidity with substance misuse in children and teenagers. Drug Alcohol Depend.

[75] Kandel DB, Johnson JG, Bird HR, Weissman MM, Goodman SH, Lahey BB (1999). Psychiatric comorbidity among adolescents with substance use disorders: findings from the MECA study. J Am Acad Child Adolesc Psychiatry.

[76] Bukstein OG, Glancy LJ, Kaminer Y (1992). Patterns of affective comorbidity in a clinical population of dually diagnosed adolescent substance abusers. J Am Acad Child Adolesc Psychiatry.

[77] Lee S (2006). Propensity score adjustment for as a weighting scheme for volunteer panel web surveys. J Official Statistics.

[78] Hu LT, Bentler PM (1999). Cutoff criteria for fit indexes in covariance structure analysis: conventional criteria versus new alternatives. Struct Equ Model.

[79] Nunnally JC, Bernstein IH (1994). Psychometric Theory.

[80] Woodside AG, Beretich TM, Lauricella MA (1993). A meta-analysis of effect sizes based on direct marketing campaigns. J Interactive Marketing.

[81] Price RA, Frank RG, Cleary PD, Goldie SJ (2011). Effects of direct-to-consumer advertising and clinical guidelines on appropriate use of human papillomavirus DNA tests. Med Care.

[82] Cohen J (1988). Statistical Power Analysis for the Behavioral Sciences.

